# Intercostal leiomyoma in a child: review of the literature

**DOI:** 10.11604/pamj.2017.28.283.14274

**Published:** 2017-11-30

**Authors:** Kleanthis Anastasiadis, Chrysostomos Kepertis, Ioannis Efstratiou, Evgenia Babatseva, Ioannis Spyridakis

**Affiliations:** 1 Department of Pediatric Surgery, Aristotle University of Thessaloniki, General Hospital “Papageorgiou”, Thessaloniki, Greece; 2 Head of Pathology Department “Papageorgiou Hospital” Thessaloniki, Greece; 3 Department of Neonatal Intensive Care, Aristotle University of Thessaloniki, General Hospital “Papageorgiou”, Thessaloniki, Greece

**Keywords:** Leiomyoma, intercostal space, chest wall, child

## Abstract

Leiomyomas of the chest wall are very rare. In a review of the current literature twelve cases were found, of which only one concerns of an intercostal leiomyoma of the chest wall. We report a case of 1 year old male child with intercostal leiomyoma who presented with a painless rigid swelling of the right chest wall. The radiological control revealed a solid mass in the right anterior sixth intercostal space. En-bloc excision of the mass by abrading of the sixth rib through right anterior thoracotomy was performed. Histopatological analysis showed a localized intercostal leiomyoma. The patient has a close follow-up for 6 months without evidence of recurrence. This is the first case of a primary intercostal leiomyoma in a child which was excised totally without reconstruction of the chest wall.

## Introduction

Leiomyomas can occur in any body parts where smooth muscles are present. As far as concern the thorax is a very atypical side for the tumor origin [1-7]. Only 12 cases of leiomyoma of the pleura have been published in the current literature [1-10]. This is the second case of primary intercostal leiomyoma, but the first described in a child.

## Patient and observation

**Figure 1:**
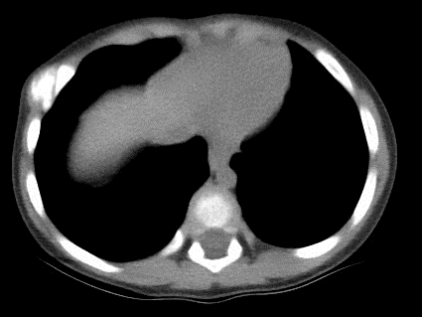
Horizontal view of contrast: enhanced computed tomography CT with a well-circumscribed solitary tumor with clear limits and calcified structure measuring 30 x 13mm in the right anterior 6^th^ intercostal space

**Figure 2:**
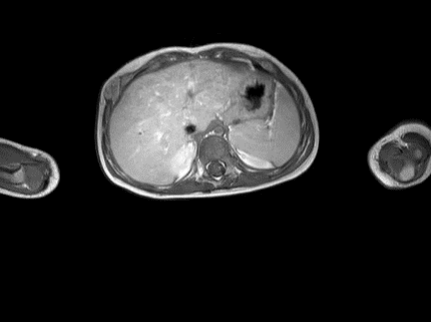
MRI showing the location of the tumor in the right anterior 6^th^ intercostal space. The structure did not show enrichment and seemed to have a wide connection with the arc of the 6^th^ rib and slightly contact with extrapleural chest wall. No infiltration of the lugs or adjacent ribs was described

**Figure 3:**
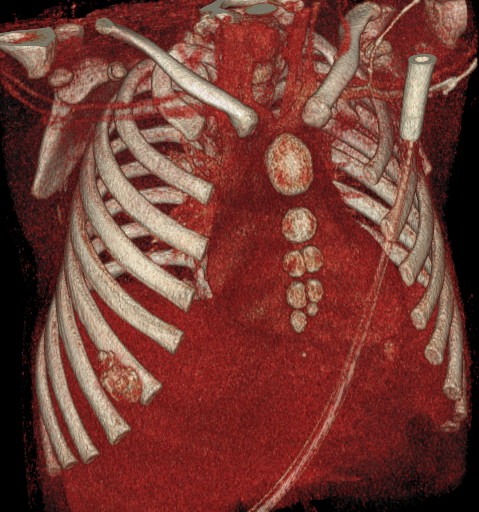
Frontal 3D (three-dimension) CT with a well-circumscribed solitary tumor with clear limits and calcified structure measuring 30 x 13mm in the right anterior 6^th^ intercostal space. The structure did not show enrichment and seemed to have a wide connection with the arc of the 6^th^ rib and slightly contact with extrapleural chest wall. No infiltration of the lugs or adjacent ribs was described

**Figure 4:**
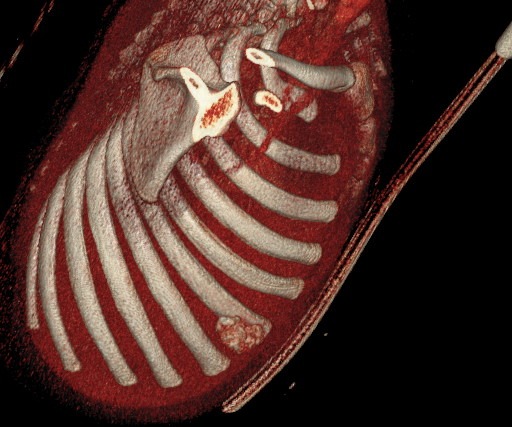
Right anterior oblique view of 3D (three-dimension) CT with a well-circumscribed solitary tumor with clear limits and calcified structure measuring 30 x 13mm in the right anterior 6^th^ intercostal space. The structure did not show enrichment and seemed to have a wide connection with the arc of the 6^th^ rib and slightly contact with extrapleural chest wall. No infiltration of the lugs or adjacent ribs was described

**Figure 5:**
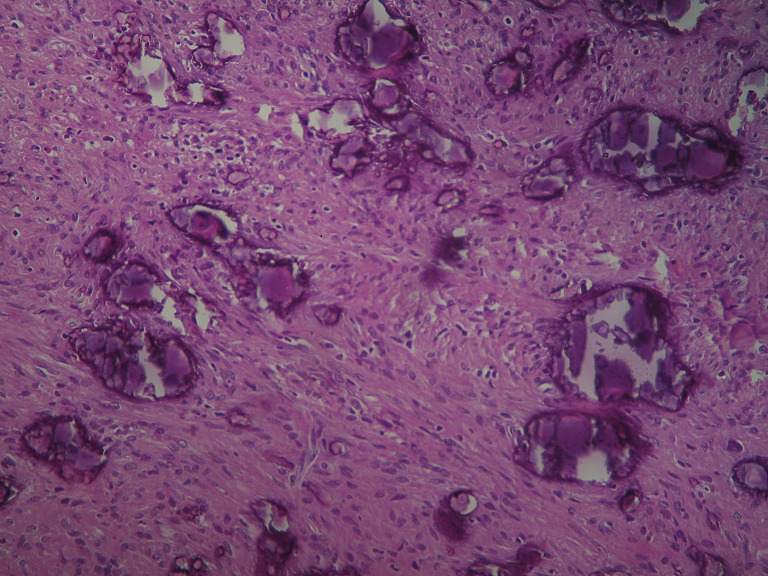
Histology of leiomyoma of deep soft tissue with calcifications. HE x200

**Figure 6:**
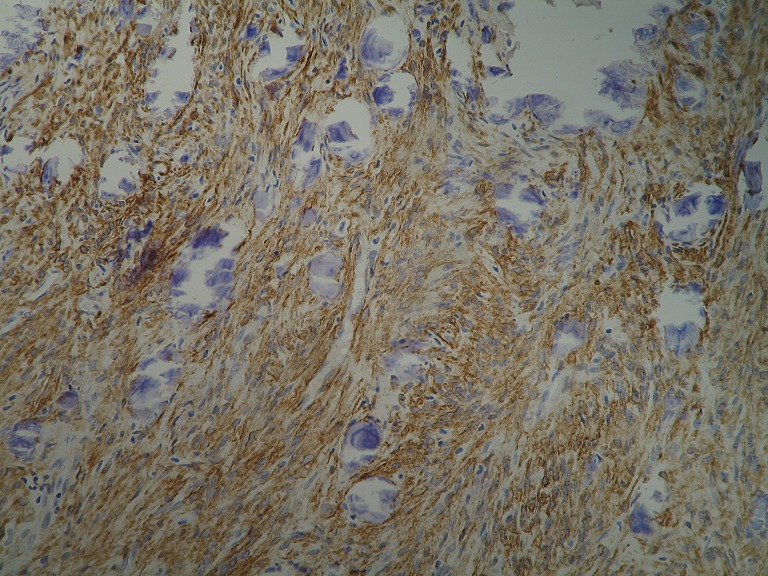
Fascicles of smooth muscle cells express smooth muscle actin (SMA) immunostatin x200

**Figure 7:**
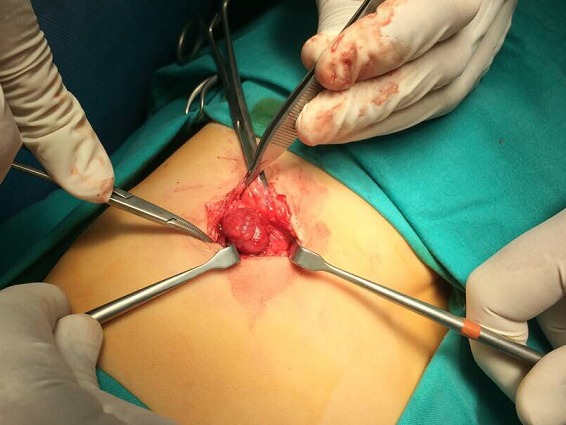
Operative findings

An 1 year old male child was admitted in the Pediatric Surgery Department complaining of painless rigid swelling of the right chest persisting for one month. Physical examination was completely normal. Laboratory blood tests did not reveal any abnormalities. Chest X-Ray did not visualize the lesion at the right hemithorax. Pathology was confirmed with computed tomography and magnetic resonance which revealed a well-circumscribed solitary tumor with clear limits and calcified structure measuring 30 x 13mm in the right anterior 6^th^ intercostal space. The structure did not show enrichment and seemed to have a wide connection with the arc of the 6^th^ rib and slightly contact with extrapleural chest wall. No infiltration of the lugs or adjacent ribs was described (Figure 1, Figure 2, Figure 3, Figure 4). A benign tumor was suspected and differential diagnosis included calcified hematoma, osteochondroma, desmoids tumor, malignant lesion. Histopathological examination of hematoxylin and eosin-stained specimens showed proliferating spindle cells with cigar-shaped nuclei. Tumor issue was free from atypia and necrosis. Immunohistochemistry revealed positive staining of the tumor cells for smooth muscle actin and negative for S-100. Tumor issue index for cell proliferation Ki-67 had a low score between 2-3% (Figure 5, Figure 6). The patient underwent a right anterior thoracotomy at the level of the 6^th^ rib of the chest wall. The encapsulated mass was excised en-bloc by abrading from the rib. Also the mass was in slightly contact with the extrapleural space (Figure 7). The child had a smooth post-operative hospital stay and was discharged from the clinic in the 3^rd^ day. He has a close follow up for 6 months without signs of recurrence.

## Discussion

Leiomyoma is a benign smooth muscle tumor which can occur in any organ but the most common site is the uterus, small bowel and esophagus [4]. Rarely originates from respiratory tract and pleura. Until now there are only twelve cases of primary pleural leiomyomas reported in the literature [1-10]. Table 1 summarizes patient and tumor characteristics of the 12 previously described cases. Routine blood and radiologic findings cannot provide a definitive diagnosis of these tumors. The origin of the previously reported eleven cases was the pleura. Only in the case No 10 (Table 1) the leiomyoma grew mainly in the intercostal space like our case. The patient was 28 years old woman versus 1 year old male child in our case. The mass in the first case was completely resected with a reconstruction of the chest wall as opposed to our own case in which en-bloc excision of the mass was performed by abrading the 6^th^ rib. A definitive diagnosis of leiomyoma must include the presence of smooth muscle fibers without signs of malignancy by hematoxylin and eosin stained specimens [1, 10]. In our case histopathology study revealed a leiomyoma of deep soft tissue with calcifications and proliferating spindle cells with cigar-shaped nuclei and the presence of fascicles of smooth muscle cells express smooth muscle actin (SMA). Differential diagnoses include osteochondrom, desmoid tumor, spindle cell carcinoma, malignant mesothelioma and other soft tissue tumors from the adjacent regions [9]. Although leiomyomas are benign they may have a low but definite malignant potential. May increase in size with local spreading to the mediastinum and may not be possible totally excised [2]. Surgical excision and long term follow-up is recommended to avoid serious symptoms or degeneration into malignant tumors. The prognosis is good for patients in whom this particular kind of tumor has been completely resected.

**Table 1:** Published case reports of primary leiomyoma of chest wall

Case	Author	Age/Sex	Clinical Symptoms	Tumor Size (cm)	Tumor Site	Tumor Origin	Procedure	Clinical Course
1	Proca DM	32 y/M	Asymptomatic	4.3x7.0	Pleura	No Detail	CR	12 M w/o rec
2	Tanaka T	40 y/F	Asymptomatic	3,5x3.0	Pleura	VSM	CR	17M w/o rec
3	Moran CA	21 y/F	Asymptomatic	Multiple Fragments	Pleura	VSM	Too Large For CR	4M w/o rec
4	Moran CA	23 y/F	Asymptomatic	10.0x9.0x5.5	Pleura	VSM	Too Large For CR	6M w/o rec
5	Nose N	55 y/F	Asymptomatic	1.5x1.5	Pleura	VSM	CR	26M w/o rec
6	Turhan K	50 y/F	Chest Pain	4x4	Pleura	VSM	CR	53M w/o rec
7	Rodriguez PM	48 y/F	Chest Pain	18x14x11	Pleura	VSM	CR	18M w/o rec
8	Ziyade S	33 y/F	Chest Pain	5.3x4.0x3.4	Pleura	VSM	CR	14M w/o rec
9	Qiou X	45 y/M	Chest Pain	9x6x5	Pleura	VSM	CR	15M w/o rec
10	Nakada MD	28 y/F	Chest Pain	4.2x3.3x3.2	Intercostal space	No Detail	CR	2M w/o rec
11	Arikura J	52 y/F	Chest Pain	6.5x5.5	Pleura	No Detail	CR	Unknown
12	Haratake N	42 y/F	Chest Pain	15x12	Pleura	Not Clear	CR	60M w/o rec
13	Current Case	12 m/ M	Asymptomatic	3.0x1.3	Intercostal Space	No Detail	CR	6M w/o rec

y :years, m: months; VSM: vascular smooth muscle; CR: complete resection; w/o rec: without recurrence, M: male, F: female

## Conclusion

We report the first case of a primary intercostal leiomyoma in a child which was excised totally without reconstruction of the chest wall. Leiomyomas when located in atypical regions are generally benign and can be totally resected. A close follow-up of the patient is recommended.

## Competing interests

The author declare no competing interests.
